# The Efficacy of a Group Cognitive Behavioral Therapy for War-Affected Young Migrants Living in Australia: A Cluster Randomized Controlled Trial

**DOI:** 10.3389/fpsyg.2016.01641

**Published:** 2016-10-31

**Authors:** Chew S. Ooi, Rosanna M. Rooney, Clare Roberts, Robert T. Kane, Bernadette Wright, Nikos Chatzisarantis

**Affiliations:** ^1^Faculty of Health Sciences, School of Psychology and Speech Pathology, Curtin UniversityPerth, WA, Australia; ^2^Faculty of Medicine, Dentistry and Health Sciences, School of Psychiatry and Clinical Neurosciences, University of Western AustraliaPerth, WA, Australia; ^3^Faculty of Health, Engineering and Science, School of Nursing and Midwifery, Edith Cowan UniversityPerth, WA, Australia

**Keywords:** mental health, children and adolescents, PTSD, depression, war trauma, cognitive behavioral therapy, school intervention, group intervention

## Abstract

**Background:** Preventative and treatment programs for people at risk of developing psychological problems after exposure to war trauma have mushroomed in the last decade. However, there is still much contention about evidence-based and culturally sensitive interventions for children. The aim of this study was to examine the efficacy of the Teaching Recovery Techniques in improving the emotional and behavioral outcomes of war-affected children resettled in Australia.

**Methods and Findings:** A cluster randomized controlled trial with pre-test, post-test, and 3-month follow-up design was employed. A total of 82 participants (aged 10–17 years) were randomized by school into the 8-week intervention (*n* = 45) or the waiting list (WL) control condition (*n* = 37). Study outcomes included symptoms of post-traumatic stress disorder, depression, internalizing and externalizing problems, as well as psychosocial functioning. A medium intervention effect was found for depression symptoms. Participants in the intervention condition experienced a greater symptom reduction than participants in the WL control condition, *F*_(1, 155)_ = 5.20, *p* = 0.024, partial η^2^ = 0.07. This improvement was maintained at the 3-month follow-up, *F*_(2, 122)_ = 7.24, *p* = 0.001, partial η^2^ = 0.20.

**Conclusions:** These findings suggest the potential benefit of the school and group-based intervention on depression symptoms but not on other outcomes, when compared to a waiting list control group.

**Trial Registration:** Australian New Zealand Clinical Trials Registry ACTRN12611000 948998.

## Introduction

Much literature on the well-being of children directly or indirectly affected by war-related trauma has consistently recorded the presence of post-traumatic stress disorder (PTSD) and other disorders (Barenbaum et al., 2004; Attanayake et al., 2009). However, there is still ambiguity surrounding the percentage of children who develop PTSD resulting from exposure to war trauma. The rate and severity vary widely depending on variables including but not limited to age, methodology, country of origin, exposure profile, and stages of flight. Whilst PTSD appears to be the primary psychological symptoms reported, depression and anxiety are commonly observed. For example, Bronstein and Montgomery (2011) systematically reviewed 22 recent epidemiological studies and found 19–54% of the children scored above the clinical cutoff score for PTSD. In addition, the rate of depression ranged from 3 to 30%.

The well-being of war-affected children has also been measured using psychosocial functioning indicators. In comparing the functioning of resettled refugee children in the United Kingdom (age 5–18 years) with ethnic minority and mainstream children, Fazel and Stein (2003) used the teacher-rated Strengths and Difficulties Questionnaire (SDQ) (Goodman et al., 2000). The total difficulties and impact scores suggest that more refugees than either ethnic minorities or mainstream children fell in “caseness” category. These findings are consistent with of the study of Leavey et al. (2004) which compared the functioning of migrant/refugee children resettled in London with local children. Results showed that more migrant/refugee children (8%) fell in the “high need” category compared with local children (4%).

Other researchers, however, have found that children are still able to carry out daily functioning and responsibilities despite clinical levels of internalizing and externalizing problems. In their survey of 182 Cambodian adolescents (aged 12–13 years) living in a Thai refugee camp, Mollica, Poole, Son, Murray, and Tor (Mollica et al., 1997)found that despite parents' ratings indicating clinical ranges for internalizing and externalizing problems, 50% of the adolescents regularly attended school. Similarly, in their study of Israeli children who have been exposed to recurrent armed conflicts, Pat-Horenczyk et al. (2007) found that 20% exhibited functional impairment in at least one area of their life but only 7.6% reported probable PTSD.

These studies suggest that the effects of traumatic experiences on PTSD and functional impairment are asymmetric, and children with PTSD symptoms may continue to function as those without PTSD symptoms. The need to comprehensively examine the clinical markers related to PTSD symptoms as well as psychosocial functioning is imperative.

Cognitive-behavioral therapy (CBT) is one of the approaches that have been studied extensively with trauma survivors, including children with PTSD resulting from earthquakes (Goenjian et al., 1997; Giannopoulou et al., 2006), hurricanes (Chemtob et al., 2002), motor vehicle accidents (Stallard et al., 1998), and sexual abuse (King et al., 2000; Cohen et al., 2004). Murray, Davidson, and Schweitzer (Murra et al., 2008) advocated for the implementation of CBT with traumatized refugee children because of its skill-oriented and time-limited nature.

Indeed, both individual and group-based CBT interventions have been employed with traumatized refugee children. In a controlled study, Layne et al. (2001) evaluated the efficacy of a group-based trauma and grief-focused intervention for 55 post-war Bosnian adolescents (aged 15–19 years) with clinical scores of PTSD, depression, and grief. The intervention spanned 20 sessions and comprised psychoeducation, cognitive restructuring, therapeutic exposure, relaxation skills, and problem-solving skills. The results indicated significant reductions of PTSD, depression, and grief symptoms from pre-test to post-test (approximately 6 months later). Layne et al. (2008) replicated this study in a randomized controlled trial (RCT) and compared the treatment program with a psychoeducational and skill-based program. Children in both conditions improved significantly in PTSD and depression symptom reduction from pre-test to 4-month follow-up. A large effect size for PTSD symptoms was found at post-test in the treatment condition, compared with a medium effect size in the comparison condition. Effect sizes for depression were small across both conditions.

A culturally sensitive CBT-based group intervention trialed in Uganda with 202 internally displaced persons (aged 15–56 years) was the EMPOWER program (Sonderegger, 2006; Sonderegger et al., 2011). The program was developed to help war-affected persons overcome their traumatic experiences through teaching emotional resiliency and reconciliation. Consisting of 13 2-h sessions, participants were assigned into either intervention or waiting list control condition according to the refugee camp in which they resided. Assessments were conducted at pre-test, post-test, and 3-month follow-up using locally developed measures. Small to large intervention effects were found for anxiety and depression symptoms, and prosocial behavior. However, the impact on PTSD symptoms was not examined. Given that the sample consisted mainly of older adolescents and adults, generalizing these results to young children should be exercised with caution. The results nevertheless show that group CBT program may be used to improve the emotional and behavioral outcomes of individuals affected by war trauma.

The efficacy of another CBT group-based intervention on war-affected children (aged 11–14 years) was evaluated by Barron et al. (2013). Teaching Recovery Techniques (TRT) (Smith et al., 2000) was implemented with these children who lived in a Palestinian region with ongoing violence. The intervention was delivered in Arabic over five sessions, each of 1.5 h duration. Participants were randomized by class into the intervention or waiting list control conditions. Close to 60% of participants reported a clinical level of PTSD at screening. Pre-test assessment was conducted 2–4 weeks prior to program delivery and post-test assessment was conducted 2 weeks after program completion. Intervention participants reported greater improvements in symptoms of PTSD, depression, grief, impact on school performance, and mental health difficulties compared with control condition participants. Girls reportedly had significantly higher grief scores than boys but it was unclear whether gender and class effects were included as a moderator in the analyses.

Barron et al. (2013) study was replicated by Qouta et al. (2012) using a larger RCT with 482 Palestinian children (aged 10–13 years) in Gaza. Contrary to the previous study, Qouta et al. found significant gender and risk effects on the outcomes. Specifically, the intervention effect was observed only in girls with low peritrauma but peritrauma did not affect the outcomes for boys. There was a significantly lower proportion of nonclinical cases at post-test among intervention participants compared with those in the control condition. Boys in both conditions did not differ significantly in their PTSD symptoms at post-test. At 6-months follow-up, no significant results were found. The intervention effects appeared modest compared with the previous study. However, these studies used different statistical methods, different age groups, and a post-war environment (ongoing violence vs. peace) which may have contributed to the different outcomes.

Ehntholt et al. (2005) used the TRT (Smith et al., 2000) in a school-based intervention aimed at reducing PTSD among resettled refugee children in the United Kingdom. Participants (aged 11–15 years) were recruited from two schools and were referred by teachers based on their previous disclosure of trauma exposure and current distress displayed in school. Baseline screening indicated that the majority of participants had experienced multiple war-related trauma and 90% of the children reported PTSD symptoms in the clinical range. A control group design was employed with 15 children placed in the intervention condition and 11 children in the waiting list control condition. A large intervention effect was observed. Children in the intervention condition reported a significant PTSD symptom reduction; the same was not reported for those in the control condition. Small but non-significant interventions effects for anxiety and depression scores were observed. In addition, a small but significant intervention effect for teacher-rated total difficulties and emotional problems was reported. However, these improvements were not maintained at 2-months follow-up. The findings were limited by the small sample size, large attrition rate, short follow-up, and absence of random allocation. Treatment integrity was not reported. In addition, given that students in each school were split into intervention and control conditions, the possibility of contamination of the intervention effect could not be discounted.

The efficacy of CBT interventions for war-exposed children was further discussed in a recent review by Rolfsnes and Idsoe (2011). They identified 19 studies which evaluated the efficacy of school-based interventions designed to reduce not only symptoms of PTSD in children but also produce an estimated medium effect size. Of eight studies conducted with war-affected children, the majority (7 out of 8) applied CBT-based interventions and reported a large effect size for PTSD symptoms. However, depression symptoms were evaluated in only some refugee studies and the effect sizes across studies were less consistent, with only one study reporting a large effect size.

Despite CBT interventions for childhood PTSD is relatively well evaluated with methodologically rigorous trials (e.g., Smith et al., 2007), empirical studies involving war-affected children, however, are lacking. For example, the CBT group-based TRT (Smith et al., 2000) specifically designed to reduce PTSD symptoms in children post-exposure to war has only been tested in three controlled trials: one in the United Kingdom (Ehntholt et al., 2005) and two with locals in Palestine (Qouta et al., 2012; Barron et al., 2013). Furthermore, Ehntholt et al.'s study was the only one investigating effects for migrant youth who have experienced war trauma but was limited by low participant number, the absence of long-term follow-up, and randomized control elements.

The Criteria for Evaluating Treatment Guidelines (American Psychological Association, 2002), defines treatment efficacy as the extent to which an intervention effect can be attributed to a given intervention as compared to no treatment or an alternative treatment. The evaluation of treatment efficacy is a pressing issue because numerous evaluation studies involving war-affected children lack a methodologically sound comparison group or rely on convenience samples for group formation. The current study addresses these identified gaps. The hypotheses of this study included:

**H1**. Participants in the intervention condition will report a significantly greater improvement in symptoms of PTSD, depression, internalizing and externalizing behavior, and psychosocial functioning from pre-test to post-test compared with participants in the waitlist (WL) control condition.
**H2**. The significant improvements observed at post-test in the intervention condition will be maintained or enhanced at 3-month follow-up.
**H3**. After receiving the intervention, participants will report a significant improvement in symptoms of PTSD, depression, internalizing, and externalizing behavior, and psychosocial functioning from immediate pre-test to immediate post-test.

## Methods

### Design

A cluster RCT design with pre-test, post-test, and 3-month follow-up was employed. The multilevel design was appropriate because participants were nested within schools (Campbell et al., 2004). Recruitment for the study was completed prior to allocating participants and schools to the conditions. To improve group equivalence, schools were match-paired by school type (public vs. private) and school level (primary vs. secondary school). The schools were not perfectly matched on socioeconomic status or on total number of students due to the limited number of participating schools. Each school in a pair was randomly allocated into either the intervention or WL control condition using a computer generated random number by the statistical supervisor of this study (RK) who was not at all involved in the clinical aspects of this study. Of the 11 schools recruited, five participated twice, producing eight school pairs (16 groups) in total (Table 1).

**Table 1:** **Total number of school by school type and school level (participant number in parentheses) in intervention and WL control conditions**.

		**Intervention *n* = 8 (45)**	**WL Control *n* = 8 (37)**
School Type	Private	2 (6)	2 (5)
	Public	6 (39)	6 (32)
School Level	Primary	2(17)	2 (18)
	Secondary	6 (28)	6 (19)

### Ethical approval

Ethical approval was received from the Curtin University Human Research Ethics Committee in July 2008 and the trial was registered with the Australian New Zealand Clinical Trials Registry (ACTRN12611000948998, https://www.anzctr.org.au/Trial/Registration/TrialReview.aspx?ACTRN=12611000948998) in September 2011. Register was completed after enrolment of participants had begun in March 2010 due to slow recruitment and delay in identifying a register. The authors confirm that all ongoing and related trials for this intervention conducted by the authors are registered.

### Participants

**Figure 1:**
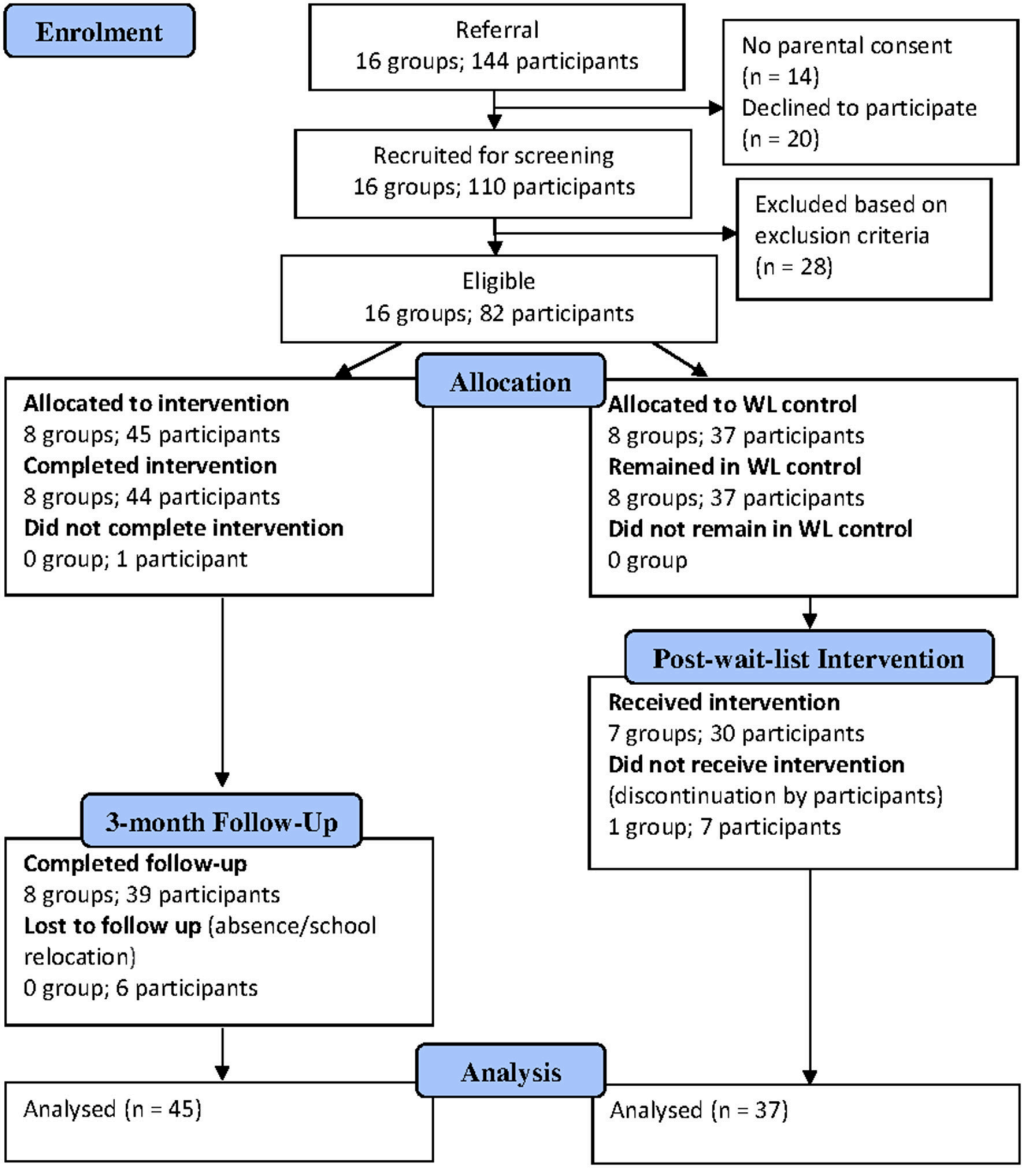
**Participant flowchart (CONSORT Flowchart)**.

An a priori power analysis was conducted to determine the sample size required to detect a small to moderate interaction effect (*f* = 0.15) between group (intervention, control) and time (pre-test, post-test) at an alpha-level of 0.05. According to G^*^Power (Faul et al., 2007), this estimate is 90 participants (to achieve power of 0.80 *P* = 0.80). A total of 82 participants (45 in intervention; 37 in WL control) were recruited from 11 schools in the Perth metropolitan areas. Inclusion criteria were self-report exposure to war or violence, living in Australia for less than 7 years and a mild to moderate level of PTSD indicated by a score between 4 and 38 on the UCLA PTSD Reaction Index for DSM-IV (UCLA PTSD Index; Rodriguez et al., 1999). Exclusion criteria were a clinical level of PTSD indicated by a score of 38 and above on the UCLA PTSD Index, limited English proficiency as determined by participants' teachers and assessors, being an unaccompanied humanitarian entrant, and currently receiving psychological treatment. Potential participants with clinical level of PTSD were excluded because the TRT (Smith et al., 2000) was not designed for this population and they were referred to relevant service providers. In cases where there was a discrepancy between parents and students, exposure reported by either party was accepted. Six participants in the intervention condition were lost at the 3-month follow-up because they were absent from school or no longer attending the schools at the time of follow up. One WL control group (*n* = 7) declined the intervention after the waiting period because of school timetable issue. Figure 1 shows participant flow, at individual and cluster level, through each stage of the study.

### Measures

The outcome measures included the Children's Revised Impact of Event Scale (CRIES-13) (Smith et al., 2003), Birleson Depression Self-Rating Scale (DSRS) (Birleson, 1981), Hopkins Symptom Checklist-37 for Adolescents (HSCL-37A) (Bean et al., 2007), and Strengths and Difficulties Questionnaire (SDQ) (Goodman et al., 2000). The internal consistency of these measures in this study was satisfactory, except the SDQ which has a Cronbach's alpha of 0.45 (prosocial score) and 0.54 (total difficulties score). The UCLA PTSD Index (Rodriguez et al., 1999) was used as a screening measure. A description of these measures is in Appendix A in Supplementary Material. A demographic form was also used to collect background information.

### Intervention

The TRT (Smith et al., 2000) is a group-based CBT program developed for survivors of war or conflicts aged 8 years or older. It is a psycho-social-education intervention, aimed at educating children about their symptoms and teaching adaptive coping strategies which include creating self-coping statements, relaxation, and exposure strategies. It was not designed for treatment purposes but rather to “prevent the need for later treatment: children who have learned and practiced the techniques contained here will be less likely to need specialist treatment services in the future” (p. 4). The sessions were organized in such a way that addresses all three elements of PTSD: intrusion, arousal, and avoidance (further information about the program may be available from the Children and War Foundation). Although it was designed to be completed in five 2-h sessions, it was delivered in eight 60-min sessions in this study because the duration of each period in schools is 60 min. The first session involved an introduction and setting of group rules whereas the second and third sessions focused on intrusion symptoms. The subsequent sessions (sessions 4 and 5) focused on arousal and the last three sessions (sessions 6–8) targeted avoidance symptoms.

### WL control condition

Participants in the WL control condition did not receive any forms of intervention during the waiting period but were offered the intervention at completion of the trial. Arrangements were made for participants who became distressed during the waiting period to be withdrawn from the program for immediate intervention. None of the participants withdrew during this period.

### Procedure

#### Recruitment

Participants were recruited between 16.3.2010 and 24.5.2011, with the last date of follow-up on 17.11.2011. Potential participants were referred by their school based on their family background and current functioning. Consent was obtained first from parents, then from the children. Parents were approached using several strategies, including holding parent information sessions at school, sending a brief or a full Participant Information letter to parents, and direct teacher-parent telephone calls. A brief information form outlining the program objective, selection criteria, and potential benefits was sent to families through schools. The form was translated into the main languages spoken by the targeted families, including Arabic, Farsi, Kirundi, Karen, and Burmese. The majority of parents responded to the brief information letter and were contacted by assessors for full consent. Telephone interpreters were used at the parent's request or assessor's discretion. The voluntary nature of the study was emphasized and written consent was sought from parents. Verbal consent was accepted for parents who do not write. Students whose parents consented were approached at school during school hours. They were screened in groups of three to five students by trained assessors (four psychology students and the primary author) after written consent was obtained. The screening assessment took approximately 60 min and was conducted in English.

### Administration of assessments and intervention

**Figure 2:**
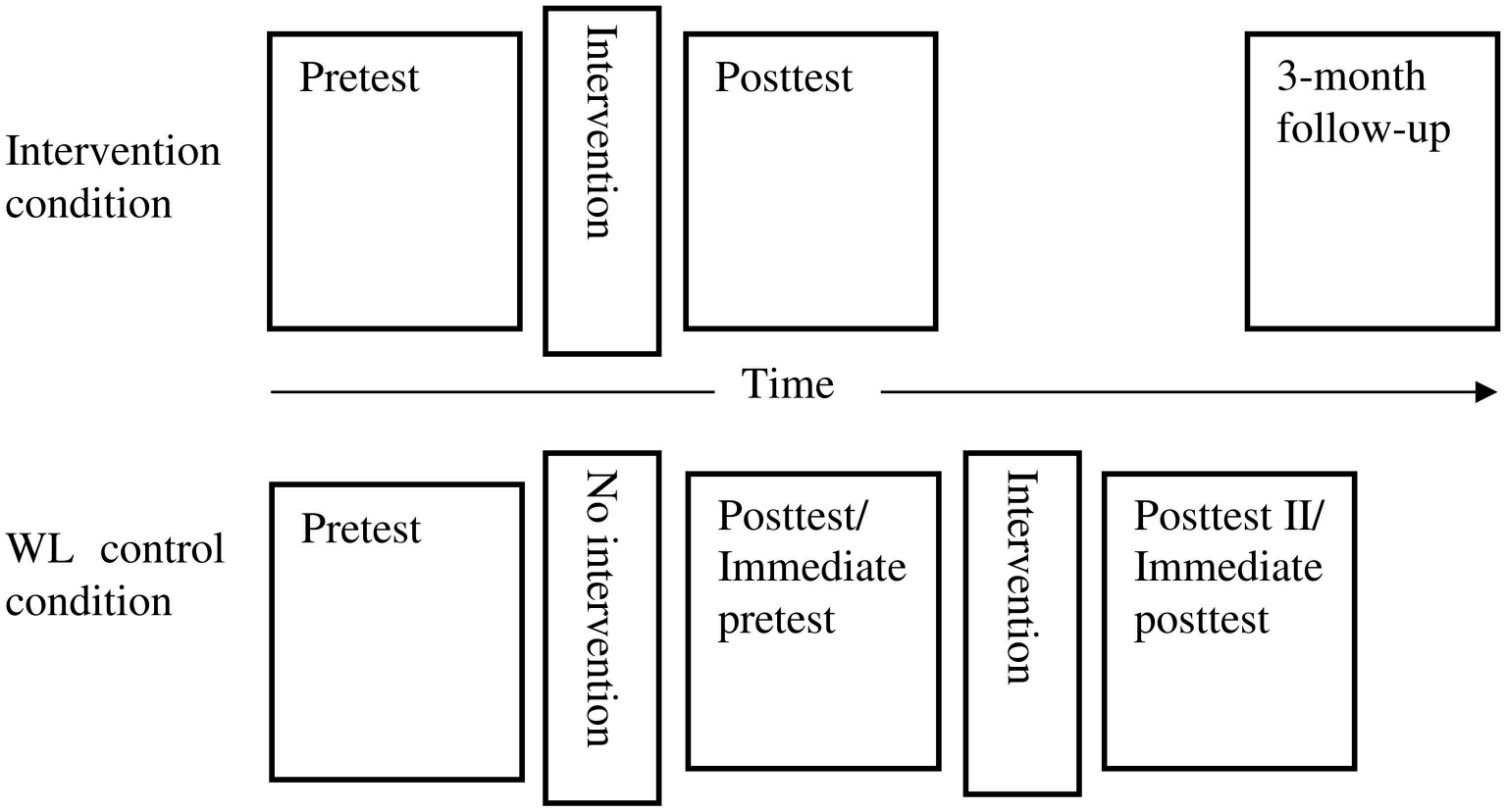
**Graphic illustration of the study procedure**.

Pre-test and post-test were conducted concurrently across both conditions 1–2 weeks before and after the intervention or waiting period. Three-month follow-up was conducted only with participants in the intervention condition because participants in the WL control condition were offered the intervention immediately after the waiting period. Another post-test (referred as WL's post-test II hereafter) was conducted with participants in the WL condition after they had received the intervention. The study procedure is illustrated in Figure 2. The intervention groups were run during school hours with group size ranging from 4 to 10 participants. The timing of the sessions was dictated by the schools. The intervention sessions were facilitated by two facilitators, comprising the primary author and a co-facilitator. The four co-facilitators were masters and PhD level psychology students at Curtin University who had received a 1-day training from the primary author. Some groups were co-facilitated by onsite school psychologists due to unavailability of trained facilitators. The primary author had received a 3-day training on program implementation by Professor William Yule and Dr Atle Dygrove in Oslo in 2009. The primary author also received ongoing clinical supervision from the project supervisors (RR and CR).

## Data analysis

A series of Generalized Linear Mixed Models (GLMMs)—one for each of the six outcome measures—were tested in order to determine whether the intervention group report changes on the outcome measures relative to the control group. The GLMMs were implemented through SPSS's (Version 20) GENLINMIXED procedure. The GLMM represents a special class of regression model. The GLMM is “generalized” in the sense that it can handle outcome variables with markedly non-normal distributions; the GLMM is “mixed” in the sense that it includes both random and fixed effects. Each of the present GLMMs included two nominal random effects (participant and school), one nominal fixed effect (group: intervention and control), one ordinal fixed effect (time: pre, post, and—for the intervention group only—3-month follow-up), and the Group × Time interaction. In order to optimize the likelihood that the GLMM solution would converge, a separate GLMM model was tested for each outcome. Within groups of conceptually similar outcomes, therefore, Bonferroni adjustments were made to the per-test alpha-levels in order to control the inflation of the family-wise error rate associated with conducting multiple statistical tests.

The traditional ANOVA repeated measures model assumes normality, homogeneity of variance, and (when the repeated measures factor has more than two levels) sphericity. The GLMM “robust statistics” option will accommodate violations of normality and homogeneity of variance. Violations of sphericity will be accommodated by changing the covariance matrix from the default of compound symmetry to autoregressive. Finally, by specifying the multilevel nature of the current data (student nested within school) in the GLMM syntax, GLMM can accommodate intra-school dependencies in the outcome measures, which were potentially problematic (ICC > 0.02; Murray and Hannan, 1990; Donner and Klar, 1996). Left uncontrolled, as in the ANOVA repeated measures model, the school effect would be expected to inflate the Type 1 error rate for the intervention effect. The a priori sample size estimate for the ANOVA model—which doesn't control for the school effect—should therefore be larger than the sample size required to give the GLMM—which does control for the school effect—the same level of power. The GLMM sample size estimate reported above (*N* = 90) was therefore based on the ANOVA model. Because we estimated the GLMM sample size using the ANOVA model, we therefore estimated GLMM effect sizes with ANOVA partial η^2^ (values of 0.01,0.06, and 0.14 reflect “small,” “medium,” and “large” effect size respectively (Cohen, 1988).

When data are collected longitudinally, we have the problem of participant attrition (wave non-response). As illustrated in the participant flow chart, six participants in the intervention condition were lost to 3-month follow-up. Wave non-response will normally reduce statistical power. Compared to the traditional statistical procedures for analyzing behavioral change (e.g., repeated measures ANOVA), GLMM is less sensitive to participant attrition because it does not rely on participants providing data at every assessment point; the GLMM maximum likelihood procedure is a full information estimation procedure that uses *all* the data present at *each* assessment point. This reduces sampling bias and the need to replace missing data. GLMM is able to use the data present at each assessment point this because time (pre, post, 3m) is interpreted as a Level 1 variable that is nested within participants at Level 2 (which is itself nested within school at Level 3).

Meaningful changes in individual participants were estimated using reliable change (RC) using PTSD and depression scores because both disorders are commonly reported by war-affected children (Bronstein and Montgomery, 2011). RC refers to reliable changes that are not due to measurement errors (Jacobson and Truax, 1991). Jacobson and Truax (1991) suggested that an RC of 1.96 or greater toward the direction of healthy range indicates *improvement*; whereas an RC of 1.96 or greater toward the direction of clinical range indicates *deterioration*. Scores that fall within 1.96 are classified as *unchanged*. Chi-square was conducted to compare RC in the intervention and WL control condition.

## Results

### Participant characteristics

The sample comprised more males (*n* = 53) than females (*n* = 29) with the majority reported being born in an African region (Table 2). Although, recruitment targeted children aged between 11 and 17 years, several children whose ages fell outside this were included because of the widely known conflicting reports of age among refugee children due to reasons such as inaccurate birthdates (Ellis et al., 2008).

**Table 2:** **Baseline characteristics for participants in the intervention (*n* = 45) and WL control (*n* = 37) conditions**.

**Characteristics**	**Intervention (*n* = 45)**			**WL Control (*n* = 37)**			**Statistics**
	***n* (%)**	***M*(*SD*)**	**Range**	***n*(%)**	***M*(*SD*)**	**Range**	
**SEX**							
Male	33 (73)			20 (54)			χ^2^_(1, *n* = 82)_ = 3.30, *p* = 0.069
Female	12 (27)			17 (46)			
**AGE**		13.13 (1.50)	10–16		12.05 (1.75)	10–17	*t*_(80)_ = 0.22, *p* = 0.826
**YEARS IN AUSTRALIA**		2.36 (1.73)	1–7		2.32 (1.87)	1–7	*t*_(75)_ = 0.09, *p* = 0.926
**ENGLISH FLUENCY**							
Good	8 (19)			11 (30)			χ^2^_(1, *n* = 79)_ = 1.23, *p* = 0.268
Fair	34 (81)			26 (70)			
**EXPOSED TO WAR**							
Yes	21 (51)			17 (46)			χ^2^_(1, *n* = 78)_ = 0.22, *p* = 0.642
No	20 (49)			20 (54)			
**SPENT TIME IN CAMPS**							
Yes	25 (61)			25 (68)			χ^2^_(1, *n* = 78)_ = 0.37, *p* = 0.544
No	16 (39)			12 (32)			
Years in camps		7.04 (3.93)	1–13		8.85 (3.29)	2-14	*t*_(44)_ = −1.69, *p* = 0.098
**NUMBER OF TRAUMAS**		4.29 (2.26)	0–8		4.17 (1.84)	1-9	*t*_(69)_ = 0.24, *p* = 0.808
**UCLA PTSD INDEX**		23.11 (8.77)	4–36		17.55 (9.05)	4-37	*t*_(80)_ = 2.82, *p* = 0.006
**BIRTH REGION**							
Africa	27(60)			19 (51)			χ^2^_(2, *n* = 78)_ = 4.68, *p* = 0.096
Asia	5 (11)			13 (35)			
Middle East	9 (20)			5 (14)			

Sample size varies across variables due to missing data. UCLA PTSD Index = UCLA PTSD Reaction Index for DSM-IV. Africa = Burundi, Congo, Guinea, Ethiopia, Kenya, Sierra Leone, Sudan, Tanzania, and Uganda; Asia = Burma, Thailand, and Sri Lanka; Middle East = Afghanistan, Iran, and Iraq.

### Outcomes

The descriptive data is presented in Table 3. It should be noted that the number of participants varies in each analysis due to missing data.

**Table 3:** **Means and standard deviations of the outcome measures for intervention (*n* = 45) and WL control condition (*n* = 37) across time**.

**Outcomes (Measures)**	**Pre-test** ***M*****(*****SD*****)**		**Post-test** ***M*****(*****SD*****)**		**Intervention's 3-month follow-up *M*(*SD*)**	**WL Control's post-test II *M*(*SD*)**
	**Intervention**	**WL Control**	**Intervention**	**WL Control**		
PTSD (CRIES-13)	23.02 (10.51)	17.92 (11.86)	15.88 (9.58)	15.68 (8.84)	12.71 (10.24)	14.17 (11.06)
Depression(DSRS)	10.96 (5.26)	9.17 (4.61)	8.68 (5.48)	8.81 (4.80)	8.29 (4.46)	8.03 (5.13)
Internalizing behavior (HSCL-37A Internalizing)	40.17 (9.58)	38.13 (7.75)	36.54 (8.52)	35.00 (8.29)	33.39 (8.60)	34.62 (8.99)
Externalizing behavior (HSCL-37A Externalizing)	15.31 (2.92)	14.67 (2.58)	15.35 (3.74)	14.30 (2.08)	14.31 (2.07)	13.93 (1.81)
Psychosocial functioning (SDQP Total Difficulties)	7.34 (3.64)	7.53 (4.24)	5.83 (2.81)	5.34 (4.03)	5.31 (3.64)	4.04 (3.05)
Prosocial behavior (SDQP Prosocial)	8.66 (1.62)	8.34 (1.60)	8.66 (1.33)	8.50 (2.00)	8.60 (1.67)	9.21 (0.79)

Sample size varies across measures and time due to missing data.CRIES-13 = Children's Revised Impact of Event Scale; DSRS = Birleson Depression Self-Rating Scale; HSCL-37A = Hopkins Symptom Checklist-37 for Adolescents; SDQP = parent-rated Strengths and Difficulties Questionnaire.

#### Comparison between the intervention and WL control group from pre-test to post-test

For the CRIES-13, a non-significant Time × Group interaction were observed, *F*_(1, 154)_ = 3.09, *p* = 0.081, partial η^2^ = 0.04, and a statistically significant time effect with a large effect size, *F*_(1, 154)_ = 8.69, *p* = 0.004, partial η^2^ = 0.14, were observed, suggesting an improvement in both conditions (Table 4). On the other hand, a statistically significant Time × Group interaction effect with a medium effect size was obtained for the DSRS, *F*_(1, 155)_ = 5.20, *p* = 0.024, partial η^2^ = 0.07. The significant interaction effect was further analyzed using LSD *post-hoc* comparisons which demonstrated a statistically significant reduction of the DSRS in the intervention condition, *t*_(155)_ = 3.84, *p* < 0.001, but not in the WL control condition, *t*_(155)_ = 0.47, *p* = 0.643. A statistically significant time effect with a large effect size was observed on the SDQP Total Difficulties, *F*_(1, 155)_ = 10.62, *p* = 0.001, partial η^2^ = 0.18, suggesting an improvement on psychosocial functioning regardless of treatment condition. All other effects were non-significant. Intercept and variance parameters for the GLMMs are reported in Table 5.

**Table 4:** **Comparisons of intervention and WL control conditions on the outcomes from pre-test to post-test**.

**Outcomes (Measures)**	**Conditions**	***n***	**Main effect of time**	**Partial η^2^**	**Main effect of group**	**Partial η^2^**	**Interaction effect**	**Partial η^2^**
PTSD (CRIES-13)	Intervention	43	*F*_(1, 154)_ = 8.69^**^	0.14	*F*_(1, 154)_ = 1.14	0.02	*F*_(1, 154)_ = 3.09	0.04
	Control	37						
Depression (DSRS)	Intervention	44	*F*_(1, 155)_ = 8.76^**^	0.11	*F*_(1, 155)_ = 0.05	0.01	*F*_(1, 155)_ = 5.20^*^	0.07
	Control	37						
Internalizing behavior	Intervention	44	*F*_(1, 155)_ = 1.83	0.10	*F*_(1, 155)_ = 0.01	0.01	*F*_(1, 155)_ = 0.03	0.00
(HSCL-37A Internalizing)	Control	36						
Externalizing behavior	Intervention	44	*F*_(1, 155)_ = 0.22	0.00	*F*_(1, 155)_ = 0.99	0.03	*F*_(1, 155)_ = 0.25	0.01
(HSCL-37A Externalizing)	Control	36						
Psychosocial functioning	Intervention	38	*F*_(1, 155)_ = 10.62^**^	0.18	*F*_(1, 155)_ = 0.06	0.00	*F*_(1, 155)_ = 0.28	0.00
(SDQP Total Difficulties)	Control	32						
Prosocial behavior	Intervention	38	*F*_(1, 155)_ = 0.00	0.00	*F*_(1, 155)_ = 1.49	0.01	*F*_(1, 155)_ = 0.07	0.00
(SDQP Prosocial)	Control	32						

*Sample size varies across measures and time due to missing data. CRIES-13 = Children's Revised Impact of Event Scale; DSRS = Birleson Depression Self-Rating Scale; HSCL-37A = Hopkins Symptom Checklist-37 for Adolescents; SDQP = parent-rated Strengths and Difficulties Questionnaire. Robust standard errors were used in analysis with the HSCL-37A Internalizing, HSCL-37A Externalizing, and SDQP Prosocial scores. The HSCL-37A Externalizing and SDQP Total Difficulties scores were analyzed within school and school type clusters*.

* *p < 0.05*.

** *p < 0.01*.

**Table 5:** **Parameter estimates for the Generalized Linear Mixed Model**.

**Measure**	**Estimate**	**95% CI**		***p*-value**
		**Lower**	**Upper**	
**PTSD (CRIES-13)**				
Intercept	15.35	10.77	19.92	< 0.001
Variance (intercept)	36.47	18.51	71.84	0.004
Variance (residual)	59.35	42.09	83.70	< 0.001
**DEPRESSION (DSRS)**				
Intercept	9.47	7.13	11.80	< 0.001
Variance (intercept)	13.34	8.49	20.98	< 0.001
Variance (residual)	8.20	5.96	11.28	< 0.001
**INTERNALIZING BEHAVIOR (HSCL-37A INTERNALIZING)**				
Intercept	36.28	32.67	39.90	< 0.001
Variance (intercept)	29.18	16.59	51.32	0.001
Variance (residual)	33.00	23.33	46.69	< 0.001
**EXTERNALIZING BEHAVIOR (HSCL-37A EXTERNALIZING)**				
Intercept	14.39	14.25	14.53	< 0.001
Variance (intercept)	2.47	1.12	5.45	0.013
Variance (residual)	5.47	3.97	7.53	< 0.001
**PSYCHOSOCIAL FUNCTIONING (SDQP TOTAL DIFFICULTIES)**				
Intercept	5.52	3.68	7.35	< 0.001
Variance (intercept)	4.76	2.29	9.88	0.007
Variance (residual)	7.48	5.24	10.67	< 0.001
**PRO-SOCIAL BEHAVIOR (SDQP PRO-SOCIAL)**				
Intercept	8.14	7.16	9.11	< 0.001
Variance (intercept)	0.59	0.21	1.64	0.054
Variance (residual)	1.60	1.10	2.31	< 0.001

CRIES-13 = Children's Revised Impact of Event Scale; DSRS = Birleson Depression Self-Rating Scale; HSCL-37A = Hopkins Symptom Checklist-37 for Adolescents; SDQP = parent-rated Strengths and Difficulties Questionnaire.

#### Changes in the intervention group from pre-test to 3-month follow-up

Participants in the intervention condition reported significant reductions from pre-test to 3-month follow-up with a large effect size on the CRIES-13, *F*_(2, 120)_ = 14.03, *p* < 0.001, partial η^2^ = 0.25, DSRS, *F*_(2, 122)_ = 7.24, *p* = 0.001, partial η^2^ = 0.20, and SDQP Total Difficulties, *F*_(1, 105)_ = 81.72, *p* < 0.001, partial η^2^ = 0.17 (Table 6). There was no further reduction of the DSRS from post-test to 3-month follow-up, *t*_(122)_ = 0.77, *p* = 0.443. An interesting finding was the significant reductions of the HSCL-37A Internalizing, *t*_(121)_ = 2.47, *p* = 0.015, and the Externalizing, *t*_(122)_ = 12.51, *p* < 0.001, from post-test to 3-month follow-up possibly suggesting a delayed intervention effect. However, this conclusion cannot be established due to the absence of a control group at follow-up.

**Table 6:** **Significance testing for intervention condition from pre-test to 3-month follow-up**.

**Outcomes (Measures)**	**Pre-test–Post-test**			**Pre-test-3-month follow-up**			**Post-test-3-month follow-up**			**Simple main effect of time**	**Partial η^2^**
	**Contrast estimate**	**SE**	**95% CI**	**Contrast estimate**	**SE**	**95% CI**	**Contrast estimate**	**SE**	**95% CI**		
PTSD (CRIES-13)	7.71^**^	2.68	[2.40, 13.02]	10.78^***^	2.15	[6.52, 15.03]	3.07	1.67	[−0.23, 6.36]	*F*_(2, 120)_ = 14.03^***^	0.25
Depression (DSRS)	2.33^**^	0.74	[0.87, 3.80]	2.91^**^	0.83	[1.27, 4.55]	0.58	0.75	[−0.91, 2.06]	*F*_(2, 122)_ = 7.24^**^	0.20
Internalizing behavior (HSCL-37A Internalizing)	1.22	3.03	[−4.78, 7.23]	4.30	3.69	[−3.00, 11.61]	3.08^*^	1.25	[0.61, 5.55]	*F*_(2, 121)_ = 3.22^*^	0.15
Externalizing behavior (HSCL-37A Externalizing)	0.07	0.80	[−1.51, 1.65]	1.13	0.71	[−0.28, 2.54]	1.06^***^	0.09	[0.90, 1.23]	*F*_(1, 122)_ = 2.54	0.06
Psychosocial functioning (SDQP Total Difficulties)	1.57	1.26	[−0.92, 4.06]	2.13^***^	0.24	[1.66, 2.60]	0.56	1.02	[−1.46, 2.58]	*F*_(1, 105)_ = 81.72^***^	0.17
Prosocial behavior (SDQP Prosocial)	−0.04	0.13	[−0.30, 0.23]	0.05	0.27	[−0.48, 0.57]	0.08	0.29	[−0.49, 0.65]	*F*_(2, 105)_ = 0.05	0.01

*Contrast estimate = estimated means difference. CI = confidence interval. SE = standard errors. CRIES-13 = Children's Revised Impact of Event Scale; DSRS = Birleson Depression Self-Rating Scale; HSCL-37A = Hopkins Symptom Checklist-37 for Adolescents; SDQP = parent-rated Strengths and Difficulties Questionnaire. Robust standard errors were used in analysis with the CRIES-13, HSCL-37A Internalizing, HSCL-37A Externalizing, SDQP Total Difficulties, and SDQP Prosocial scores. The HSCL-37A Externalizing and SDQP Total Difficulties scores were analyzed within school type and school clusters*.

* *p < 0.05*.

** *p < 0.01*.

*** *p < 0.001*.

#### Comparison between the intervention and WL control group from immediate pre-test (intervention's pre-test; WL control's post-test) to immediate post-test (intervention's post-test; WL control's post-test II)

Given that participants in the WL control condition were offered the intervention after the waiting period, their pre- and post-intervention changes were compared with those of the intervention condition. A statistically significant but small Time × Group interaction effects were observed for the CRIES-13, *F*_(1, 140)_ = 7.86, *p* = 0.006, partial η^2^ = 0.04, and DSRS, *F*_(1, 142)_ = 5.19, *p* = 0.024, partial η^2^ = 0.04 (Table 7). LSD comparisons revealed a significant reduction in the CRIES-13 in the intervention condition, *t*_(140)_ = 3.37, *p* = 0.001, but not in the WL control condition, *t*_(140)_ = 1.13, *p* = 0.262. A similar pattern was observed for the DSRS, with significant reduction in the intervention condition, *t*_(142)_ = 2.24, *p* = 0.027, but not in the WL control condition, *t*_(142)_ = −0.72, *p* = 0.473. These results suggest that only children in the intervention condition reported significant reductions of PTSD and depression symptoms even though children in the WL control condition also received the same intervention. A large group effect was observed on the SDQP Total Difficulties, *F*_(1, 124)_ = 146.38, *p* < 0.001, partial η^2^ = 0.16.

**Table 7:** **Comparisons of intervention and WL control conditions on outcomes at immediate pre-test and immediate post-test**.

**Outcomes (Measures)**	**Conditions**	***n***	**Main effect of time**	**Partial η^2^**	**Main effect of group**	**Partial η^2^**	**Interaction effect**	**Partial η^2^**
PTSD (CRIES-13)	Intervention	43	*F*_(1, 140)_ = 8.44^*^	0.12	*F*_(1, 140)_ = 2.29	0.06	*F*_(1, 140)_ = 7.86^*^	0.04
	Control	40						
Depression (DSRS)	Intervention	44	*F*_(1, 142)_ = 0.78	0.08	*F*_(1, 142)_ = 0.29	0.03	*F*_(1, 142)_ = 5.19^*^	0.04
	Control	30						
Internalizing behavior	Intervention	44	*F*_(1, 142)_ = 0.01	0.01	*F*_(1, 142)_ = 0.05	0.09	*F*_(1, 142)_ = 6.58	0.02
(HSCL-37A Internalizing)	Control	30						
Externalizing behavior	Intervention	44	*F*_(1, 142)_ = 0.01	0.01	*F*_(1, 142)_ = 1.81	0.09	*F*_(1, 142)_ = 0.00	0.02
(HSCL-37A Externalizing)	Control	30						
Psychosocial functioning	Intervention	38	*F*_(1, 124)_ = 2.61	0.14	*F*_(1, 124)_ = 146.38^***^	0.16	*F*_(1, 124)_ = 0.12	0.00
(SDQP Total Difficulties)	Control	27						
Pro-social behavior	Intervention	38	*F*_(1, 124)_ = 1.78	0.01	*F*_(1, 124)_ = 0.06	0.09	*F*_(1, 124)_ = 1.71	0.02
(SDQP Prosocial)	Control	27						

*Sample size varies across measures and time due to missing data. Immediate pretest = Intervention's pretest; WL control's post-test; Immediate post-test = Intervention's post-test; WL control's post-test II. CRIES-13 = Children's Revised Impact of Event Scale; DSRS = Birleson Depression Self-Rating Scale; HSCL-37A = Hopkins Symptom Checklist-37 for Adolescents; SDQP = parent-rated Strengths and Difficulties Questionnaire. Robust standard errors were used in analysis with the CRIES-13, HSCL-37A Internalizing, HSCL-37A Externalizing, SDQP Total Difficulties, and SDQP Prosocial scores. The HSCL-37A Externalizing and SDQP Total Difficulties scores were analyzed within school type and school clusters*.

* *p < 0.05*.

*** *p < 0.001*.

### Reliable change

Reliable change (RC) was calculated using the statistics and formulae in Tables 8, 9 respectively. Table 10 displays the proportion of participants making reliable improvement, deterioration, and not making reliable changes on the CRIES-13 and DSRS. The differences in the proportion of cases making these changes were not statistically significant between the conditions, $\chi_{\left(1,\;\text{N}=76\right)}^{2}=$ 1.76, *p* = 0.414, *V* = 0.152, and $\chi_{\left(1,\;\text{N}=77\right)}^{2}=$ 2.10, *p* = 0.350, *V* = 0.165 for CRIES-13 and DSRS respectively.

**Table 8:** **Statistics used to compute reliable change using the CRIES-13 and DSRS**.

**Statistic**	**Definition**	**Value**	
		**CRIES-13**	**DSRS**
X_1_	Pre-test score of an individual		
X_2_	Post-test score of an individual		
S_diff_	Standard error of difference between the two test scores	7.19	3.48
S_E_	Standard error of measurement of the test score	5.09	2.45
S_1_	Standard deviation of the sample at pre-test	11.37	5.06
r_xx_	Reliability of the test	0.80	0.80
M_1_	Pre-test mean of the sample	20.66	10.15

Reliabilities of the CRIES-13 and DSRS were obtained from Smith et al. (2003) and Birleson (1981) respectively.

**Table 9:** **Formulae used to calculate reliable change**.

	**Reliable change**	**S_diff_**	**S_E_**
Formula	$\frac{x2\;-\;x1}{\text{Sdiff}}$	√2 (S_E_)^2^	S_1_√(1 – r_xx_)

**Table 10:** **Reliable change at post-test and 3-month follow-up for intervention and WL control conditions**.

**Outcomes (Measures)**	**Conditions**	**Post-test, n (%)**				**3-month follow-up, n (%)**			
		***n***	**I**	**NC**	**D**	***n***	**I**	**NC**	**D**
PTSD (CRIES-13)	Intervention	39	8 (21)	29 (74)	2 (5)	37	15 (41)	19 (51)	3 (8)
	Control	37	4 (11)	32 (86)	1 (3)				
Depression (DSRS)	Intervention	41	5 (12)	35 (85)	1 (2)	37	8 (22)	29 (78)	
	Control	36	2 (6)	33 (92)	1 (3)				

Sample size varies across measures and time due to missing data. I = improved; NC = no change; D = deteriorated.

### Program integrity

Program integrity was assessed using information collected from a facilitator's log completed by group facilitators after each session. The mean percentage of content covered across the groups ranged from 84 to 100% (*M* = 92.76, *SD* = 5.58), indicating relatively high adherence to the intervention manual.

## Discussion

The aim of this study was to examine the efficacy of the TRT (Smith et al., 2000) in reducing PTSD, depression, internalizing and externalizing, and improving psychosocial functioning in children exposed to war-related trauma. Although, no effect was found for PTSD symptoms, results showed that after controlling for clustering effects, the participants in the intervention condition had significantly lower depression symptoms with reliable improvement. The significant Time × Group interaction effect with a medium effect size for depression suggests that children who received the intervention had significantly greater reductions in depression symptoms from pre-test to post-test compared to children in the WL control condition. Furthermore, this improvement was maintained at 3-month follow-up. However, the hypothesis that participants in the intervention would experience a greater reduction of PTSD symptoms compared to children in the WL control condition was not confirmed.

The absence of a significant reduction of PTSD symptoms in the intervention condition compared to the WL control condition was in contrast to our hypothesis and failed to replicate results reported by Barron et al. (2013) and Ehntholt et al. (2005). This is surprising considering that within-group comparisons showed a significant symptom reduction from pre-test to follow-up. It seems that participants in the intervention condition did in fact improve after receiving the intervention but the improvement was not greater than the improvement of participants in the control condition. A probable reason may be the inadequate sample size. The current sample size (*n* = 82) was smaller than expected and hence it may not have had sufficient statistical power to detect the small (partial η^2^ = 0.04) treatment effect for PTSD symptoms observed in this study. The effect sizes reported in Barron et al. and in Ehntholt et al. (calculated using crude data) were moderate to large which is likely to have contributed to the different findings from the current study. An earlier study also observed that patients with low severity of PTSD tended to improve less than patients with high severity (Foa et al., 1995). Although, Ehntholt et al. did not appear to specifically recruit participants with clinical levels of PTSD, the PTSD pre-test scores of their participants were relatively higher compared to the scores of the current sample. Therefore, it is plausible that with higher baseline, participants in Ehntholt et al.'s study made greater gains than participants in the current study, contributing to the different findings. It is possible that the relatively short duration of the intervention may have contributed to the lack of effect whilst a longer intervention duration with subsequent increased dosage may have assisted in finding an intervention effect for PTSD. In addition, the interval between intervention and short term follow-up of 3 months where intervention effects may not have yet been apparent and the relatively low number of participants, may also have contributed to the lack of a significant finding for PTSD symptoms. Future researchers should consider a higher dose of intervention and longer term follow-up.

The medium intervention effect observed for depression suggests that the TRT (Smith et al., 2000) is effective in reducing depression symptoms in children. This is a significant finding considering the brevity of the intervention. CBT interventions have been found to effectively reduce depression symptoms in children who experience traumatic reactions in earlier studies. For example, as discussed, Layne et al. (2008) observed significant reductions in both PTSD and depression symptoms in their participants after the 17-session trauma and grief-focused intervention. Similarly, Smith et al. (2007) reported significant improvements in their participants after a 10-week CBT intervention. In comparison to these interventions, the TRT was brief and will be very useful in time- and resources-poor situations, such as schools. From school's perspective, the brief intervention will enable practitioners to provide the intervention to students needing such support within a 10-week school term before they leave the school. This is because in Western Australia, school-aged migrants with limited English literacy generally spend 1–2 years in a school that has intensive English support before enrolling in a mainstream school.

One possible explanation for effects in depression as opposed to PTSD apart from the limitations outlined above is that according to the cognitive model of PTSD, persistent negative appraisals of trauma and symptoms contribute to negative emotions and maladaptive coping strategies (Ehlers and Clark, 2000). Depression is generally believed to be caused by depressive schema that lead to negative cognitions and a reduction of negative cognitions has been found to mediate the treatment effects of CBT interventions on depression symptoms (Kaufman et al., 2005; Garratt et al., 2007). Considering that the TRT comprises cognitive re-structuring and relaxation, it is possible that these skills led to a sense of mastery, and a reduction of negative cognitions and hopelessness.

In contrast to predictions, there were no significant intervention effects for internalizing behavior and externalizing behavior outcomes. This is surprising in relation to the intervention effect for depression outcome. However, given that the internalizing subscale of the HSCL-37A measures both anxiety and depression symptoms, the finding may have been complicated by the inconsistent result between depression and PTSD outcomes. Even though there might be a probable delayed intervention effect, evidenced by the reductions in both outcomes from post-test to 3-month follow-up, no inferences could be made due to limited data.

Consistent with Giannopoulou et al. (2006) study, participants in the intervention condition had significant improvement in parent-rated psychosocial functioning at post-test. However, significant improvement was also reported for participants in the control condition. It is possible that the short study period did not cater for the time lapse between symptom reduction and functional improvement, a notion raised in earlier studies (Kazdin, 2001; Bolton et al., 2007). As a result, the potential functional improvements associated with the significant reduction of depression symptoms in the intervention condition could not be detected. Having said that, one may also argue that the finding is in line with the reduction of PTSD symptoms in both conditions. This is an interesting finding and warrants further study.

At post-intervention, contrary to our hypothesis, only participants in the intervention group reported significant reductions in PTSD and depression symptoms even though participants in the WL control group received the same intervention. Because the WL control group showed reductions in PTSD and depression symptoms during the waiting period, the failure of this group to show significant intervention effects on these outcomes might reflect floor effects. Symptomatic improvement during waiting periods in the absence of specific therapeutic intervention has been reported in the literature (Smith et al., 2007; Hardy and Stallard, 2008; Roberts et al., 2010) and this needs to be taken into account when understanding the current results and limitations.

The current study is believed to be the first to have investigated the reliable change of refugee children receiving this intervention. Results show no significant differences between children in both groups even though more children in the intervention condition made reliable improvement (21% on PTSD; 12% on depression scores), compared to children in the WL control condition (11%; 6%). Also, 41% of children in the intervention condition made reliable improvement in symptoms of PTSD at 3-month follow-up. One of the intervention studies involving war-affected children that have investigated reliable change was conducted by Layne et al. (2001). Layne et al. reported higher improvement rates in their study, with 35% of participants making a reliable improvement on depression scores, and 50% on PTSD and grief scores. However, direct comparison between our study and Layne et al. (2001) study is difficult because their study had a different target population (15–19 year-old Bosnian adolescents) and a longer treatment process (20 sessions of trauma- and grief-focused group therapy). In the same way, Jordans et al. (2010) reported a greater proportion of reliable improvement on depression scores (23%) at post-test after a 15-session intervention. It may be worthwhile for future research to investigate whether these differences are a result of dose-response relationship in therapy.

### Limitations

The study has several limitations that affect interpretation, including small sample size, intervention-waiting list control design which does not control for non-treatment specific factors, and a lack of control group at follow-up. In order to increase participation, parents were approached through information sessions, information letter, and teacher-parent phone call. In using these approaches we may have introduced a bias in that only motivated parents would participate. Given that parents were not blinded from treatment allocation, demand characteristics and social desirability could not be ruled out from the data. Moreover, the complexity of personality- and context-related factors interwoven with cultural differences of the study sample may have impacted on the results. The wide range of intervention group size at each intervention site due to recruitment difficulty may have affected the group dynamic and possibly study outcomes. Due to limited resources, it was not possible to employ blind examiners and independent checks on treatment integrity. However, efforts were made to mitigate possible biases arising from administration procedure by employing standardized assessment tools, manualized treatment protocols, and facilitators' log.

Future researchers should consider comparing the intervention with a placebo control group in order to tease out therapeutic effects from non-treatment specific factors. Future studies should also consider a longer waiting period for better comparison of follow-up data. Considering the heterogeneous nature of refugee populations, future research is needed to establish generalizability of the current findings to other ethnic, cultural, and linguistic groups. Given that our sample was restricted to tight inclusion and exclusion criteria, future studies may include children with limited English fluency or with different ethnic backgrounds. It will also be beneficial to investigate the benefits of the supplemental parent component of the TRT (Smith et al., 2000).

## Conclusion

This is the first cluster RCT of the TRT (Smith et al., 2000) in educational settings for war-affected migrant children living in Australia. This is also the first trial of the TRT to have utilized GLMM for statistical hypothesis testing. The result of this study suggests the benefit of the TRT on depression but not on PTSD symptoms, internalizing, and externalizing behaviors, and psychosocial functioning. The complexity of mental health of children affected by war trauma, diversity of interventions, and scarcity of rigorous studies warrant further studies.

## Author contributions

CO, RR, CR, RK, and BW designed the study. CO performed the study. CO and RK analyzed the data. CO, RR, CR, RK, BW, and NC prepared the manuscript and approved it to be published.

## Funding

This study was funded by Curtin University School of Psychology Ph.D. fund for CO and Western Australia Health Promotion Foundation (Healthway) http://www.healthway.wa.gov.au/ (Healthway Researcher Starter Grant No. 19923) to CR. The funders had no role in the study design, data collection and analysis, decision to publish, or preparation of the manuscript.

### Conflict of interest statement

The authors declare that the research was conducted in the absence of any commercial or financial relationships that could be construed as a potential conflict of interest.
