# Supplementary material for: The Efficacy of a Group Cognitive Behavioral Therapy for War-Affected Young Migrants Living in Australia: A Cluster Randomized Controlled Trial
Source: Front Psychol. 2016 Oct 31;7:1641. doi: 10.3389/fpsyg.2016.01641 (PMC5086861; doi:10.3389/fpsyg.2016.01641)
Supplement: Supplementary file 1 [file DataSheet1.docx]

**Appendix A**

**UCLA PTSD Reaction Index for DSM-IV**

The UCLA PTSD Reaction Index for DSM-IV (UCLA PTSD Index; Rodriguez, Steinberg, & Pynoos, 1999) is a revised version of a frequently used child PTSD instrument, Posttraumatic Stress Reaction Index ([Pynoos et al., 1987](#_ENREF_270)). It comprises 27 Yes/No items assessing trauma exposure and 22 items assessing PTSD symptoms using a 5-point Likert scale ranging from 0 (*none of the time*) to 4 (*most of the time*). It produces two scores (a) an overall severity score, ranging from 0 to 68, by summing the 17 core items; and (b) subscores for intrusion, avoidance, and arousal symptoms by summing items belonging to each subscale. Although there is limited information about the specific cutoff score for a particular trauma type or population, a cutoff score of 38 has been proposed in the literature (Steinberg, Brymer, Decker, & Pynoos, 2004). The Cronbach’s alpha of this measure has been found to be in the range of .90 (Ellis, Lhewa, Charney, & Cabral, 2006; Steinberg et al., 2004). It also has good convergent validity with the PTSD Module of the Schedule for Affective Disorders and Schizophrenia for School-Age Children, *r* = .70 (Rodriguez, Steinberg, Saltzman, & Pynoos, 2001). It was also found to correlate significantly with self-report measures of PTSD-related symptoms such as the Birleson Depression Self-rating Scale, *r* = .72 ([Ellis et al., 2006](#_ENREF_94)). It has been used with displaced and refugee children and adolescents (Ellis, MacDonald, Lincoln, & Cabral, 2008; [Morgos, Worden, & Gupta, 2007](#_ENREF_225)).

**Children’s Revised Impact of Event Scale**

The Children’s Revised Impact of Event Scale (CRIES-13; Smith, Perrin, Dyregrov, & Yule, 2003) is a 13-item PTSD scale measuring symptoms of intrusion (4 items), avoidance (4 items), and arousal (5 items). The items are rated on a 4-point Likert scale ranging from 0 (*none*), 1 (*rarely*), 3 (*sometimes*) to 5 (*a lot*), producing a total score ranging from 0 to 65. It has good internal consistency with a Cronbach’s alpha of .80. It has been found to correlate strongly with other measures of PTSD, including the PTSD Checklist, *r* =.84 (Creamer, Bell, & Failla, 2003), and the UCLA PTSD Index, *r* =.79 (Giannopoulou, Smith, Ecker, Strouthos, Daikaiakou, & Yule, 2006). It has been used with children exposed to war trauma (e.g., Sack, Seeley, Him, & Clarke, 2014; Heptinstall, Sethna, & Taylor, 2004).

**Birleson Depression Self-Rating Scale**

The Birleson Depression Self-Rating Scale (DSRS; Birleson, 1987) is an 18-item scale measuring depression symptoms in children and adolescents aged between 8 and 14 years. The measure comprises both emotional (e.g., sadness and loneliness) and behavioural components of depressive symptomatology (e.g., sleep difficulty and lack of activity). Each item is scored on a 3-point scale ranging from 0 (*never*), 1 (s*ometimes*) to 2 (*mostly*), with repressive response scoring 2 and non-depressive responses scoring 0. The total score is derived by summing all items to produce a total score ranging from 0 to 36.

Birleson (1981) reported split-half reliability coefficient of .86 and test-retest reliability of .80. The duration of test-retest was not specified in Birleson’s study but similar result (*r* = .78) was reported by Panter-Brick, Goodman, Tol, and Eggerman (2011) who conducted a seven days test-retest in an Afghanistan sample. According to the normative data obtained from a sample of British children, the DSRS has reasonably high specificity (ranging from 77% to 88%) and sensitivity (ranging from 64% to 67%; Birleson et al., 1987). It was found to correlate significantly with another depression measure, the Child Depression Index, with correlations ranging from .67 to .76 (Charman, 1994). It has been widely used to assess the well-being of refugee children (Papageorgiou et al., 2000) and to evaluate treatment efficacy (Tol et al., 2008).

**Hopkins Symptom Checklist-37 for Adolescents**

The Hopkins Symptom Checklist-37 for Adolescents (HCSL-37A; Bean, Derluyn, Eurelings-Bontekoe, Broekaert, & Spinhoven, 2007) was adapted from the Hopkins Symptoms Checklist-25 (Derogatis, LIpman, Rickels, Uhlenhuth, & Covi, 1974) and is used extensively with non-Western or refugee children. It contains 37 items assessing internalising behaviours and externalising behaviours. Only 36 items were used in the study because Item 13, which asks children if their sexual desire decreased over the last month, was omitted considering that it might be developmentally inappropriate for younger participants in our studies. Each item is answered on a 4-point Likert scale ranging from 1 (*never*), 2 (*sometimes*), 3 (*often*), to 4 (*always*), producing a total score ranging from 36 to144 (allowing for the omission of Item 13). The HSCL-37A has a Cronbach’s alpha of .90 for the total score, .92 for the internalising subtest (depression and anxiety subscales), and .75 for the externalising subtest [36]. It has a 12-month test-retest reliability of .63 for the total score, .64 for the internalising subtest, and .53 for the externalising subtest. In addition, the HSCL-37A has good construct validity, with the total and internalising scores showing significant and positive correlations with the Reactions to Traumatic Stress Checklist (correlations ranged from .66 to .79), the Stressful Life Events questionnaire (correlations ranged from .30 to .41), and the Strengths and Difficulties Questionnaire (correlations ranged from .64 to .70 ). The correlations between externalising subtest and these measures were weaker but still significant, with correlations ranging from .23 to .43. The HSCL-37A is available in 15 languages and has been used with adolescents from different cultural groups.

**Strengths and Difficulties Questionnaire**

The Strengths and Difficulties Questionnaire (SDQ; Goodman, Ford, Simmons, Gatword, & Meltzer, 2000) is a brief screening measure that provides information about children’s and adolescents’ emotional, behavioural, and relationship functioning. The parent-rated SDQ (SDQP) with impact component was employed in this study. It comprises 25 items that fall under five subscales: hyperactivity (Items 2, 10, 15, 21 and 25), emotional symptoms (Items 3, 8, 13, 16 and 24), conduct problems (Items 5, 7, 12, 18 and 22), peer relationship difficulties (Items 6, 11, 14, 19 and 23), and prosocial behaviours (Items 1, 4, 9, 17 and 20). Each item is rated on a 3-point scale, ranging from 0 (*not true*), 1 (*somewhat true*) to 2 (*very true*). Items 7, 11, 14, 21 and 25 were reversed scored. The subscale scores are obtained by summing the respective subscale items; while the total score is derived by summing all, except the prosocial subscale. For the purpose of this study, the total and prosocial scores were used. The SDQP has shown good internal consistency, with Cronbach’s alpha coefficients of .82 and .85 for the total difficulties and prosocial scores respectively (Goodman, 2001). The Cronbach’s alpha of the subscale scores were found to range from .57 to .77. The test-retest reliabilities over four to six months were found to range from .57 to .72. The SDQ is also found to correlate highly with the Child Behavior Checklist, *r* = .59 to .87, on the five subscales (Goodman & Scott, 1999). It has been widely used with refugee children and adolescents from different cultural groups (e.g., Derluyn & Broekaert, 2007; Fazel & Stein, 2013).

**References**

Bean T, Derluyn I, Eurelings-Bontekoe E, Broekaert E, Spinhoven P. Validation of the multiple language versions of the Hopkins Symptom Checklist-37 for refugee adolescents. Adolescence. 2007 Spring;42(165):51-71.

Birleson P. The validity of depressive disorder in childhood and the development of a self-rating scale: A research report. J Child Psychol Psychiatry. 1981 Jan; 22(1):73-88. doi:10.1111/j.1469-7610.1981.tb00533.x

Birleson P, Hudson I, Buchanan DG, Wolff S. Clinical evaluation of a self-rating scale for depressive disorder in childhood (Depression Self-rating Scale). J Child Psychol Psychiatry. 1987 Jan;28(1):43-60. doi:10.1111/j.1469-7610.1987.tb00651.x

Charman T. The stability of depressed mood in young adolescents: A school-based survey. J Affect Disord. 1994 Feb;30(2):109-16. doi:10.1016/0165-0327(94)90038-8

Creamer M, Bell R, Failla S. Psychometric properties of the Impact of Event Scale-Revised. Behav Res Ther. 2003 Dec;41(12):1489-96. doi:10.1016/j.brat.2003.07.010

Derluyn I, Broekaert E. Different perspectives on emotional and behavioural problems in unaccompanied refugee children and adolescents. Ethn Health. 2007 Apr;12(2):141-62. doi:10.1080/13557850601002296

Derogatis LR, Lipman RS, Rickels K, Uhlenhuth EH, Covi L. The Hopkins Symptom Checklist (HSCL): A self-report symptom inventory. Behav Sci. 1974 Jan;19(1):1-15. doi:10.1002/bs.3830190102

Ellis BH, Lhewa D, Charney M, Cabral H. Screening for PTSD among Somali adolescent refugees: Psychometric properties of the UCLA PTSD Index. J Trauma Stress. 2006 Aug;19(4):547-51. doi:10.1002/jts.20139

Ellis BH, MacDonald HZ, Lincoln AK, Cabral HJ. Mental health of Somali adolescent refugees: The role of trauma, stress, and perceived discrimination. J Consult Clin Psychol. 2008 Apr;76(2):184-93. doi:10.1037/0022-006X.76.2.184

Fazel M, Stein A. Mental health of refugee children: Comparative study. BMJ. 2003 Jul;327:134. doi:10.1136/bmj.327.7407.134

Giannopoulou I, Smith P, Ecker C, Strouthos M, Dikaiakou A, Yule W. Factor structure of the Children's Revised Impact of Event Scale (CRIES) with children exposed to earthquake. Pers Individ Dif. 2006 Apr;40(5):1027-37.

Goodman R. Psychometric properties of the Strengths and Difficulties Questionnaire. J Am Acad Child Adolesc Psychiatry. 2001 Nov;40(11):1337-45. doi:10.1097/00004583-200111000-00015

Goodman R, Ford T, Simmons H, Gatward R, Meltzer H. Using the Strengths and Difficulties Questionnaire (SDQ) to screen for child psychiatric disorders in a community sample. Br J Psychiatry. 2000 Dec;177:534-9. doi:10.1192/bjp.177.6.534

Goodman R, Scott S. Comparing the Strengths and Difficulties Questionnaire

and the Child Behavior Checklist: Is small beautiful? J Abnorm Child Psychol. 1999 Feb;27(1):17-24. doi:10.1023/A:1022658222914

Heptinstall E, Sethna V, Taylor E. PTSD and depression in refugee children: Associations with pre-migration trauma and post-migration stress. Eur Child Adolesc Psychiatry. 2004 Dec;13(6):373-80. doi:10.1007/s00787-004-0422-y

Morgos D, Worden JW, Gupta L. Psychosocial effects of war experiences among displaced children in Southern Darfur. Omega (Westport). 2007-2008;56(3):229-53. doi:10.2190/OM.56.3.b

Panter-Brick C, Goodman A, Tol WA, Eggerman M. Mental health and childhood adversitites: A longitudinal study in Kabul, Afghanistan. J Am Acad Child Adolesc Psychiatry. 2011 Apr;50(4):349-63. doi:10.1016/j.jaac.2010.12.001

Papageorgiou V, Frangou-Garunovic A, Lordanidou R, Yule W, Smith P, Vostanis P. War trauma and psychopathology in Bosnian refugee children. Eur Child Adolesc Psychiatry. 2000 Jun;9(2):84-90. doi:10.1007/s007870050002

Pynoos RS, Frederick C, Nader KO, Arroyo W, Steinberg A, Eth S, Nunez F, Fairbanks L. Life threat and posttraumatic stress in school-aged children. Arch Gen Psychiatry. 1987 Dec;44(12):1057-63. doi:10.1001/archpsyc.1987.01800240031005

Rodriguez N, Steinberg A, Pynoos RS. UCLA PTSD Index for DSM-IV instrument information: Child version, parent version, adolescent version. Los Angeles, CA: UCLA Trauma Psychiatry Services; 1999.

Rodriguez N, Steinberg AM, Saltzman WS, Pynoos RS. PTSD Index: Preliminary psychometric analyses of child and parent versions. Annual Meeting of the International Society for Traumatic Stress Studies; 2001 Dec 6-9; New Orleans, LA.

Sack WH, Seeley JR, Him C, Clarke G. Psychometric properties of the impact of events scale in traumatized Cambodian refugee youth. Int J Soc Psychiatry. 2014 Feb;60(1):6-20. doi:10.1016/S0191-8869(98)00030-0

Smith P, Perrin S, Dyregrov A, Yule W. Principal components analysis of the impact of event scale with children in war. Pers Individ Dif. 2003 Feb; 34(2):315-22. doi:10.1016/S0191-8869(02)00047-8

Steinberg AM, Brymer MJ, Decker KB, Pynoos RS. The University of California at Los Angeles Post-traumatic Stress Disorder Reaction Index. Curr Psychiatry Rep. 2004 Apr;6(2):96-100. doi:10.1007/s11920-004-0048-2

Tol WA, Komproe IH, Susanty D, Jordans MJ, Macy RD, De Jong JT. School-based mental health intervention for children affected by political violence in Indonesia: A cluster randomized trial. JAMA. 2008 Aug 13;300(6):655-62. doi: 10.1001/jama.300.6.655. doi:10.1001/jama.300.6.655
